
JOURNAL INFORMATION
==============================
NLM Title Abbreviation: ScientificWorldJournal
Journal ID: ScientificWorldJournal
NLM Journal ID: 101131163
Journal ID: TSWJ

The Scientific World Journal

ISSN: 2356-6140
EISSN: 1537-744X
Publisher: Wiley

ARTICLE INFORMATION
==============================
PMCID: PMC5956414
PMID: 29861669
DOI: 10.1100/tsw.2004.57
Article ID: 803105
Article version: 1
Subjects: Research Article

Current Indications for the Use of The Artificial Genitourinary Sphincter and Management of It's Complications

Elliott Daniel S.
Barrett David M.
Mayo Clinic, Department of Urology, Rochester, MN, USA
Academic Editor: Anthony Atala

Publication date: 2004
Electronic publication date: 2004 Jun 7
Volume: 4
First page: 114
Last page: 127
Copyright: Copyright © 2004 Daniel S. Elliott and David M. Barrett.
Copyright year: 2004
License: This is an open access article distributed under the Creative Commons Attribution License, which permits unrestricted use, distribution, and reproduction in any medium, provided the original work is properly cited.
License URL: https://creativecommons.org/licenses/by/3.0/

==============================
Article
*TheScientificWorldJOURNAL* (2004) 4 (S1), 114–127
ISSN 1537-744X; DOI 10.1100/tsw.2004.57

### Current Indications for the Use of The Artificial Genitourinary Sphincter and Management of It's Complications

Daniel S. Elliott, M.D. and David M. Barrett, M.D.
*Mayo Clinic, Department of Urology, Rochester, MN*

**E-mails**: Elliott.Daniel@mayo.edu

*Previously published in the Digital Urology Journal*

---

**DOMAIN**: urology, aging, medical care (men's health), surgery

---

### INTRODUCTION

The first artificial urinary sphincter was implanted in 1972 and since then many advances in product design, improvements of surgical techniques, and proper patient selection have resulted in decreasing complication rates and improved patient satisfaction.[1] The currently used artificial sphincter (AUS) is the American Medical Systems (AMS) 800. This prosthesis consists of silicone components: (1) an elastic pressure-balloon reservoir; (2) a control pump; and (3) an inflatable occlusive urethral cuff. The balloon reservoir is manufactured to hold a volume of fluid which exerts a range of preset pressures (51-60, 61-70, 71-80, and 81-90cm water) that can be selected from based on the site of cuff placement and the clinical situation. The pump contains a unidirectional valve, a refill-delay resistor and a deactivation button. This assembly allows for complete control of cuff activation/deactivation by the patient and physician. In the activated mode, the urethral cuff is de-pressurized by squeezing the pump, thereby, forcing fluid from the cuff through the unidirectional valve into the balloon reservoir (Fig. 1). Following a delay period of three to five minutes, time allowed for voiding, fluid automatically flows back to repressurize the cuff.

The AMS-800 can be non-surgically deactivated by pressing the deactivation button on the pump which prevents fluid in the balloon from returning to the cuff. Deactivation allows for healing to take place without compression on the urethra. Deactivation also facilitates catheterization, cystoscopy and transurethral resection, if necessary. Activation is accomplished by a sustained squeeze of the pump.

### INDICATIONS FOR AUS IMPLANTATION AND PATIENT SELECTION

The candidate for AUS implantation is the patient with urinary incontinence due to sphincteric insufficiency, with normal detrusor function and bladder compliance. Recognizing that urinary incontinence may be the result of coexisting sphincteric incompetence and bladder compliance and contractility abnormalities, AUS implantation can be considered in these patients assuming that these coexisting abnormalities can be adequately managed.

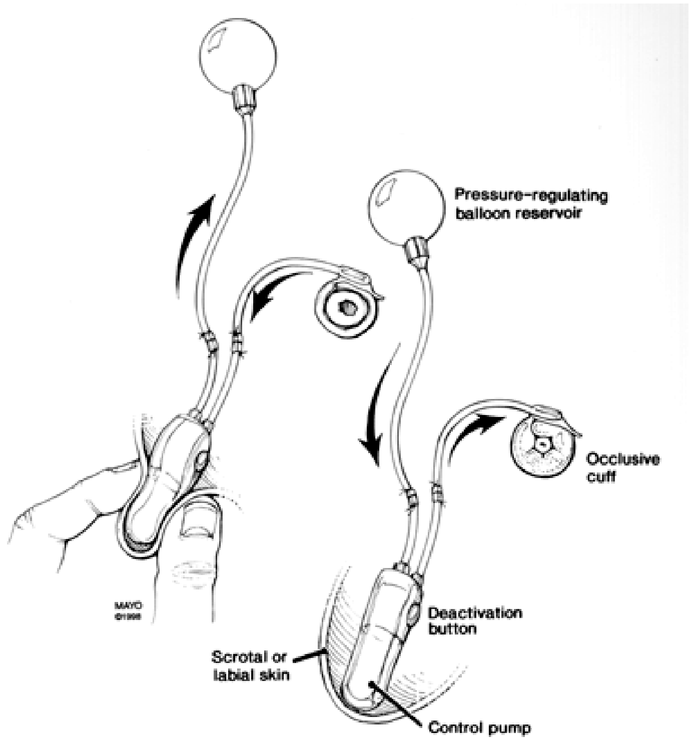

**FIGURE 1.** Components and cycling of the AS-800 AUS; pressure on the pump transfers fluid out of the cuff to the reservoir, the cuff then refills passively.

Patients with sphincteric incontinence from a variety of causes are appropriate candidates for AUS implantation. In our experience the most common etiologies for incontinence requiring AUS are following radical retropubic prostatectomy and transurethral resection of the prostate.[2] A minimum of six months is recommended between the time of prostatectomy and AUS implantation to allow time for continence to return. Sphincteric insufficiency may also develop following injury or radiation to the pelvis and spinal cord. Congenital disorders such as myelomeningocele can result in loss of sphincteric function. The bladder neck is the site of cuff placement in these patients. All women and children who are candidates for AUS implantation have cuffs placed on the bladder neck. The bladder neck is the optimal location for AUS implantation because of the thick muscular tissue and good blood supply in this area. However, following prostatectomy, the bladder neck tissue becomes scarred and fixed to the surrounding tissues, and, therefore, the bulbous urethra is the preferred site for cuff placement in this group of patients.

### PREOPERATIVE CONSIDERATIONS

Preoperative evaluation should include urine analysis and culture to assure that the urine is sterile prior to AUS implantation. Upper tract abnormalities should be evaluated with excretory urogram and/or voiding cystourethrogram if warranted by the patients history. Repair of grade two, or greater, reflux should be repaired at or before the time of AUS implantation. Bladder augmentation may be required in patients with noncompliant, low capacity bladders to make them suitable candidates for AUS implantation. Urodynamic studies are recommended in specific patient situations, however it is not mandatory. If there are concerns about bladder dysfunction then either urodynamics, or preferably a videoeurodynamics is required.

### OPERATIVE TECHNIQUE

Patients are usually admitted the morning of surgery. Patients having bladder neck cuff placement are instructed to undergo a Fleets enema in the evening prior to surgery. At the time of surgery, infection precautions cannot be overemphasized. On the morning of surgery, females should have a standard vaginal douche preparation. Broad-spectrum intravenous antibiotics, most commonly vancomycin and gentamicin, are administered in the preoperative holding area. Antibiotic prophylaxis must include coverage for Staphylococcus epidermidis and Escherichia coli, as these organism are responsible up to 80% of genitourinary prosthetic infections.[3] In the operating room strict adherence to sterile techniques are essential to minimize potential infections. A complete ten-minute surgical scrub (abdomen, external genitalia and perineum with iodophors, is undertaken immediately following the surgical shaving. Draping should allow access to the lower abdomen and perineum. A 12-French Foley catheter is inserted in all males after preparation and draping. All operating room personnel should wear surgical hoods to limit contamination of the operative field, and traffic in and out of the operating room should be limited.[2] The prosthesis should be handled only when necessary and should be isolated away from tissue, blood, and sponge and towel fibers, which could cause malfunction of the device.

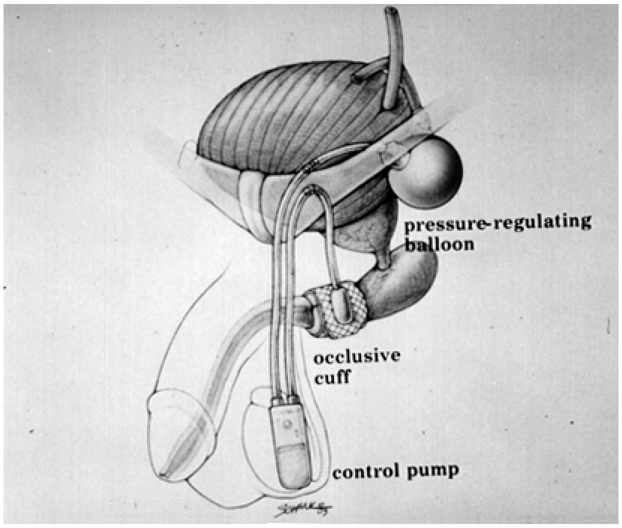

**FIGURE 2**. The position of the bulbous urethral cuff, reservoir, and pump in the adult male.

### Bulbous Urethral Cuff in the Adult Male (Fig. 2)

The patients are positioned in the dorsolithotomy position to expose the perineum. After hair removal, skin preparation and draping, a sterile 12-F Foley catheter is placed and the bladder is drained. A 4-5cm midline skin incision is made over the bulbous urethra (Fig. 3). Through the perineal incision, deeper tissues are divided and carried down until the bulbocavernosus muscle is identified. A plane is established between the bulbocavernosus muscle and the tunica albuginea of the corporal bodies circumferentially for a length of 1.5-2cm. At our institution, we preserve the bulbocavernosus muscle. Though the decision to place the cuff around the muscle is not shared by all, it is our feeling that the muscle will theoretically provide more bulk and potentially decrease the risk for atrophy and subsequent cuff erosion. This plane allows placement of the cuff around the muscular tissue, and not directly in contact with the urethral bulb, to reduce the risk of cuff erosion. Special care must be taken to avoid injury to the urethra at the 12 o'clock position where the dorsal urethra is attached to the intercorporal septum. If the urethral defect is large and/or the repair is questionable, the procedure should be abandoned

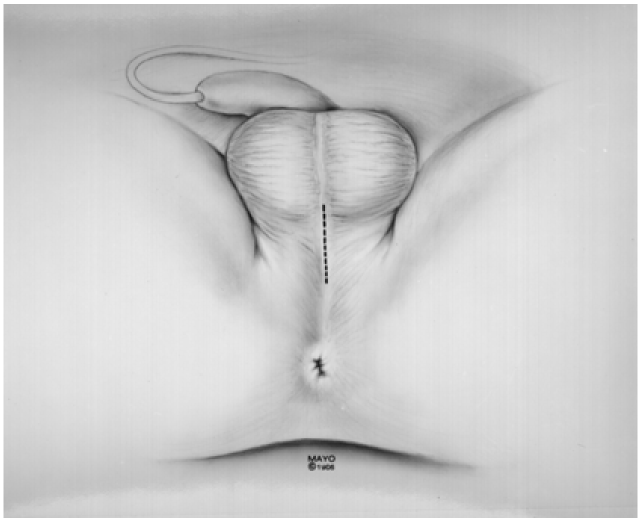

**FIGURE 3.** Site of perineal incision for placement of the bulbous urethral cuff. An incision 4-5 cm in length the median raphe along the scrotum.

and a Foley catheter left in place. If the urethral injury is small, the defect can be closed with 4-0 or 5-0 absorbable suture and a different urethral segment is chosen for cuff placement. An unrecognized urethral injury can lead to early cuff erosion.

Although cuffs of varying sizes are available, in our recent series 267 of 272 (98%) of our patients received a 4.5cm cuff around the bulbous urethra.[2] The cuff is passed tab-first behind the urethra (Fig. 4) in the direction that points toward the side of subsequent pump and reservoir placement, usually the side of hand dominance. Next, the cuff tubing is brought anteriorly and is passed through the opening in the tab, and the cuff is snapped into place using gentle opposing traction applied to the tab and tubing. The tubing now should be pointed toward the side where the reservoir will be inserted (Fig. 5). A rubber shod clamp closed to the first click is placed near the end of the tubing.

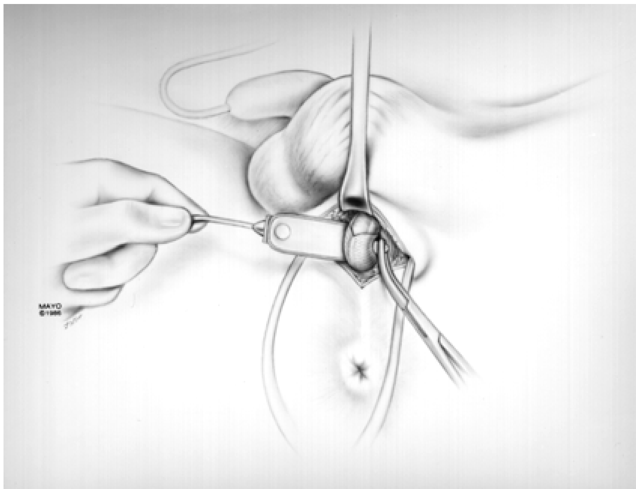

**FIGURE 4.** Passing the cuff around the bulbous urethra.

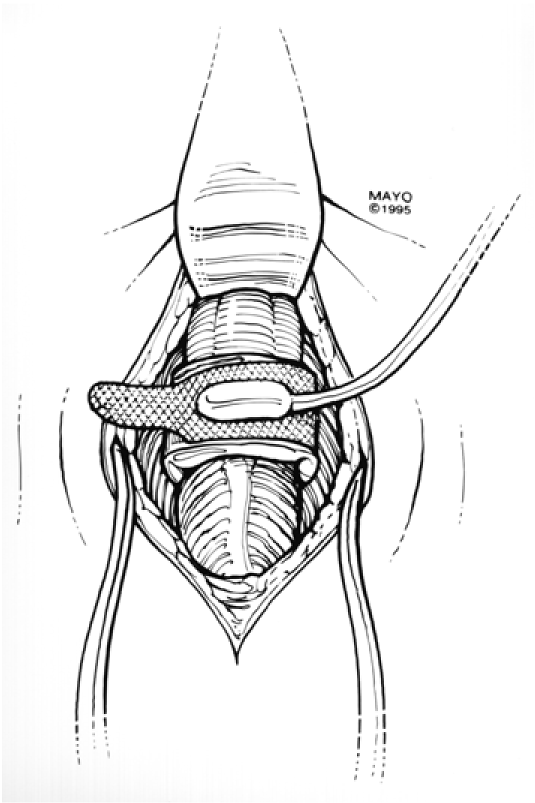

**FIGURE 5.** Completion of cuff placement around the bulbous urethra and securing in place.

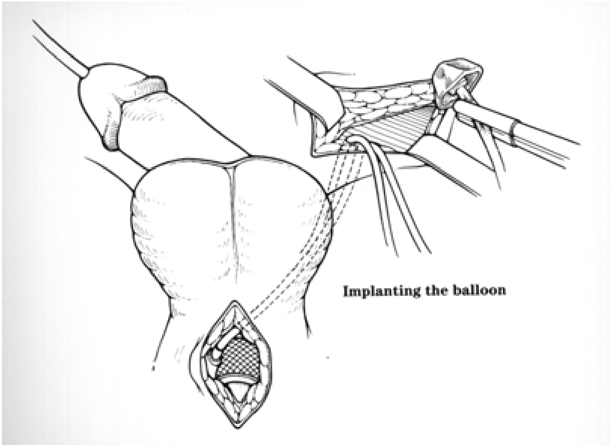

**FIGURE 6.** Placement of balloon reservoir deep to the rectus muscle.

Reservoir placement (Fig. 6) follows by making a 4-5cm transverse incision in the lower abdomen overlying the rectus sheath. The anterior rectus sheath is exposed and subsequently incised parallel to the fascia approximately 2cm. A pocket is bluntly created between the belly of the rectus muscle to allow for placement of the deflated balloon reservoir beneath the muscle in a preperitoneal location. The balloon reservoir is filled with 22cc and a shod clamp is placed near the end of the reservoir tubing, on the first click, to prevent fluid loss. It is our practice to fill the reservoir with iso-isomotic contrast medium. We feel this simplifies future diagnosis and management of patients who return in the future with recurrent urinary incontinence.

In most patients (82%) a 61-70cm water pressure balloon reservoir is used.[2] However, a lower pressure reservoir, usually a 51-60cm water pressure, is selected to minimize tissue ischemia in high risk patients such as those who have had radiation therapy or prior surgery. The fascial defect is closed with running absorbable suture after the inflated reservoir is properly positioned deep to the rectus muscle. On the ipsilateral side of the reservoir, a long clamp is passed over the pubis in the plane between Scarpa's fascia and the rectus fascia down to the perineal incision. The cuff tubing is gently grasped and guided up to the lower abdominal wound (Fig. 7).

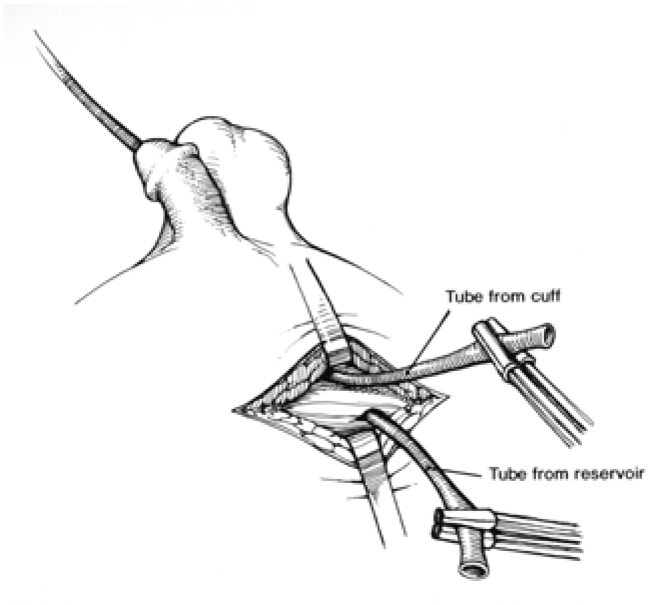

**FIGURE 7.** Positioning of cuff and reservoir tubing after placement of the balloon reservoir.

To facilitate pump placement, an ipsilateral subcutaneous pouch is created superficial to Scarpa's fascia using Hegar dilators up to #15, just as is done during bladder neck cuff placement. The pouch extends down to the most dependent portion of the hemiscrotum. The pump is placed in the pouch with the deactivation button oriented laterally so that it is palpable against the skin. To prevent proximal migration of the pump during tubing connection, a Babcock clamp is loosely placed around the pump and scrotal skin.

The appropriate tubing connections are now made. The cuff tubing is connected to the pump tubing with a right-angle connector to prevent kinking, and the reservoir tubing is connected to the pump tubing with a straight connector. Color-coded tubing has simplified this step. Tubing connections are either fastened with 2-0 prolene suture, or with the Quick-Connect set depending on the surgeon's preference. After the connections are completed, all clamps are removed from the tubing and the system is allowed to pressurize. Appropriate cycling of the device is demonstrated and then the sphincter is deactivated so that there is no urethral compression. Drains are not necessary nor recommended. The abdominal and perineal incisions are closed in layers with absorbable suture and then covered with sterile pressure(gentle) dressings.

### Bladder Neck Cuff in the Male

Male patients undergoing bladder neck cuff placement are positioned supine with their legs abducted slightly. A rectal tube is placed to aid in the identification of the rectum during bladder neck dissection. A lower midline incision is made and carried down through the rectus and transversalis fascia. The peritoneum is not entered. The retropubic space is developed bluntly. A plane is developed superior to the endopelvic fascia but distal to the posterior aspect of the bladder trigone where the ureters enter (Fig. 8,9). A plane is established between the bladder neck and proximal urethra anteriorly, and the rectum posteriorly. The width of this plane should be 2cm to accommodate the bladder neck cuff. An umbilical tape or Penrose drain is placed around the bladder neck. To identify injury to the bladder neck that may occurred during dissection, an antibiotic solution containing methylene blue is used to fill the bladder. Small tears can be closed with 3-0 or 4-0 absorbable suture. If a rectal injury is identified, the defect should be closed primarily and the sphincter implantation must be postponed. Using a calibrated measuring tape with the Foley catheter in place, the bladder neck is measured circumferentially (Fig. 10). In men the cuff size will range between 8-14cm. The Foley catheter should be removed if it is larger than 14-F. The cuff is passed posterior to the bladder neck towards the side of final pump and reservoir placement. The cuff is then snapped into place with the cuff tubing exiting laterally. The cuff tubing is then passed through the belly of the rectus muscle and the anterior rectus fascia approximately 4-5cm cephalad to the pubic symphysis on the side of future pump placement (Fig. 11). The end of the cuff tubing is clamped to the first click with a shodded clamp.

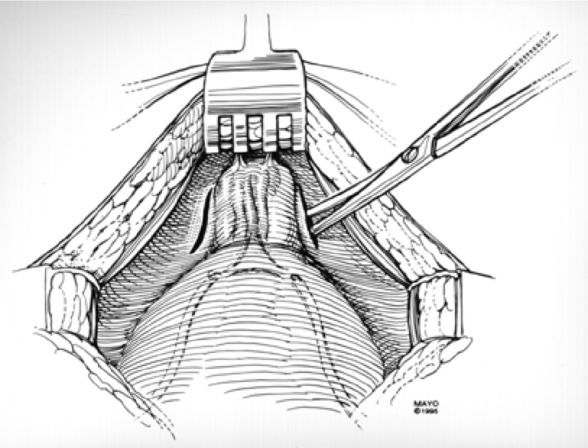

**FIGURE 8.** Dissection around the bladder neck.

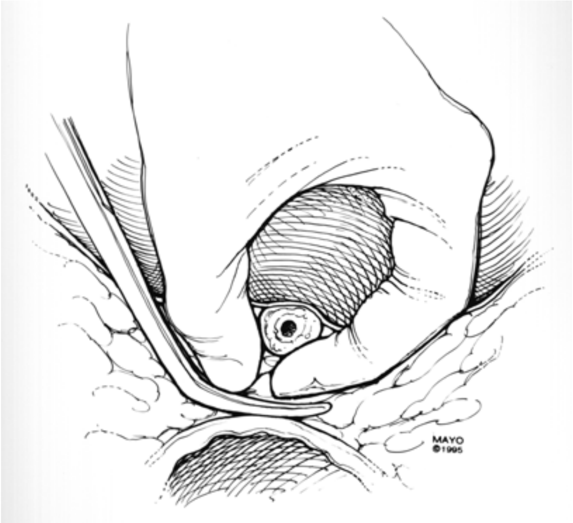

**FIGURE 9.** Developing the plane superior to the endopelvic fascia but distal to the posterior aspect of the bladder trigone.

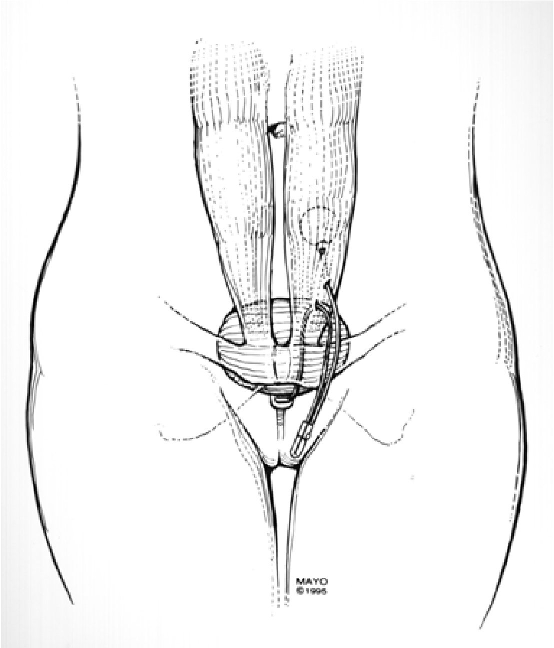

**FIGURE 10.** Location of components of the female AUS.

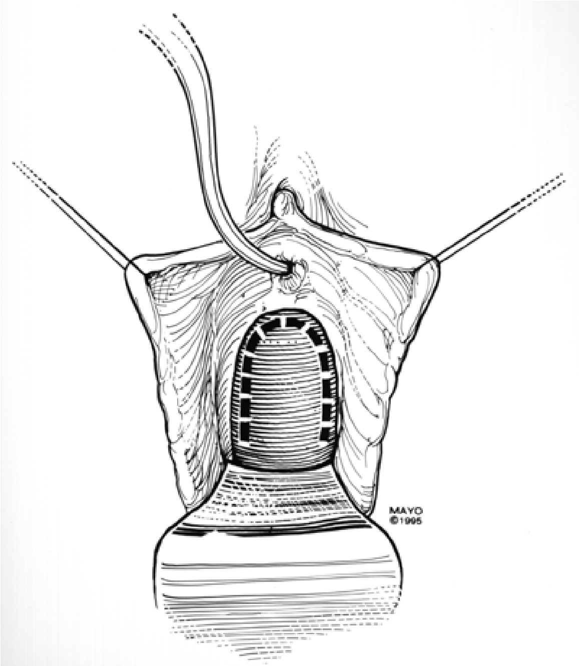

**FIGURE 11.** Site of anterior vaginal wall incision for transvaginal AUS implantation.

Balloon reservoir selection and placement follows cuff placement. The most commonly used balloon is the 61-70cm water pressure reservoir. The empty reservoir is placed in the prevesical space and the tubing is brought out through the rectus muscle and anterior fascia next to the cuff tubing (Fig. 11). Before the balloon is filled, the anterior abdominal fascia is closed in the midline with absorbable suture. The balloon is then filled with 22cc of iso-osmotic contrast medium, and the tubing is clamped at the first click with a shodded clamp.

Pump placement proceeds by creating a subcutaneous pocket in the lateral aspect of one hemiscrotum using Hegar dilators up to #15 (Fig. 11). The pump is placed in the dependent position of the scrotal pocket with the deactivation button positioned laterally for external access. Tubing connections between the pump and reservoir, and the pump and cuff are made using straight connectors tied with 2-0 prolene suture or fastened with a Quick-Connect set. The device is cycled (figure 12,13), then the cuff is deactivated. The surgical field is irrigated copiously with antibiotic solution, then the incision is closed in layers with absorbable suture.

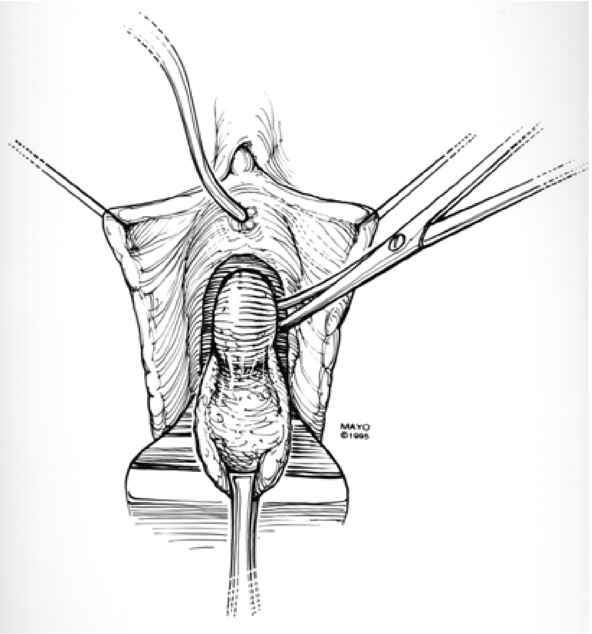

**FIGURE 12.** Dissection of vaginal wall off the bladder neck.

### AUS Implantation with a Penile Prosthesis

Urinary incontinence and impotence often coexist after radical prostatectomy or radiation therapy. A penile prosthesis and AUS can be implanted simultaneously or in staged procedures at least two to three months apart. It is recommended that the AUS be implanted first if simultaneous procedures are undertaken. The procedure can be completed through a low transverse suprapubic incision if a multicomponent penile prosthesis is chosen. A midline perineal incision is also required if the sphincter cuff is to be placed on the bulbous urethra. The AUS components should be implanted, connected, cycled and deactivated prior to introducing the penile prosthesis. The individual reservoirs are placed under their respective rectus muscle. The pumps are placed in separate subcutaneous hemiscrotal pockets.

### Bladder Neck Cuff in the Female

AUS implantation in females can be accomplished either through an abdominal or transvaginal approach. The decision to use the transvaginal approach is usually determined by the surgeon's experience with anatomy and techniques of transvaginal surgery. This review will focus on the transvaginal approach. The position of the AUS components are seen in figure 10.

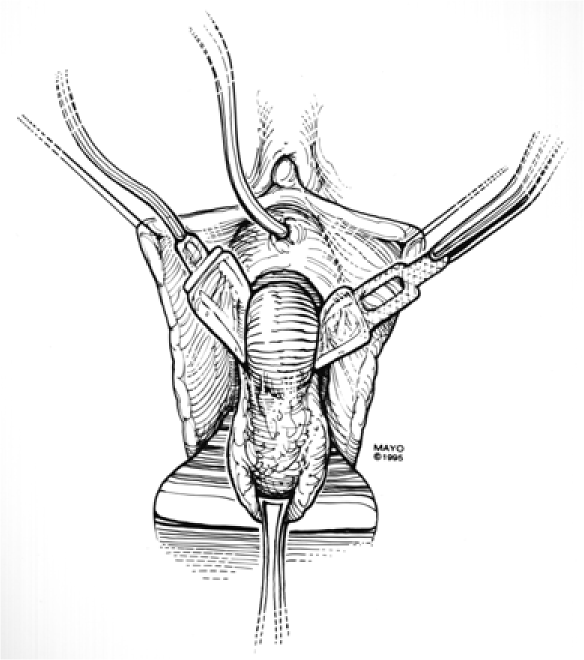

**FIGURE 13.** Cuff as it is passed behind the bladder neck transvaginally.

### Transvaginal Approach of Bladder Neck Cuff Placement

Patients are positioned in the dorsal lithotomy position. A formal iodophor preparation of the vagina is completed. The lower abdomen and perineum are included in the surgical field. A sterile 16-F Foley catheter is inserted into the bladder per urethrum. The labia minora are retracted laterally using stay sutures, and a weighted speculum is placed in the vagina. A semicircular incision is made so that the apex of the incision is midway between the urethral meatus and the bladder neck (Fig. 11). The flap of vaginal wall is dissected free from the urethra with its base toward the bladder neck (Fig. 12). The retropubic space is entered with scissors pointing toward the ipsilateral shoulder on each side of the bladder neck. The endopelvic fascia is swepped laterally off the pubic bone. Using blunt dissection, the proximal urethra and bladder neck are mobilized off the underside of the pubis. After removal of the Foley catheter, the measuring tape is placed around the bladder neck. The appropriately sized cuff is then passed posteriorly towards the side of future pump and reservoir placement (Fig. 13). The cuff is snapped into place, and the cuff tubing should be exiting laterally (Fig. 14). Cystoscopy can be used at this point to check placement of the cuff so that the cuff is distal to the ureteral orifices.

Following cuff placement, a transverse suprapubic incision is made, exposing the anterior abdominal fascia. A small midline incision is made through the fascia, thereby exposing the prevesical space. A 61-70cm water pressure balloon reservoir is placed through the fascial defect in the prevesical space. The reservoir tubing is brought through the rectus belly and fascia lateral to the midline on the side of future pump placement. The balloon is filled with 22cc of iso-osmotic contrast and a shodded clamp is placed at the end of the tubing to prevent fluid loss. The cuff tubing is transferred retropubically through the vaginal incision up into the abdominal incision. The cuff tubing should penetrate the rectus muscle and fascia near the reservoir tubing as previously illustrated (Fig. 10).

Hegar dilators are used to create a subcutaneous pocket in the labia majora. The pump is placed into the most dependent portion of the pocket so that it can be easily accessed. Connections are then made between the pump and reservoir, and the pump and cuff tubing as previously described. The abdominal and vaginal incisions are closed in layers using absorbable suture. The Foley catheter is reinserted and an antibiotic-soaked pack is placed into the vagina.

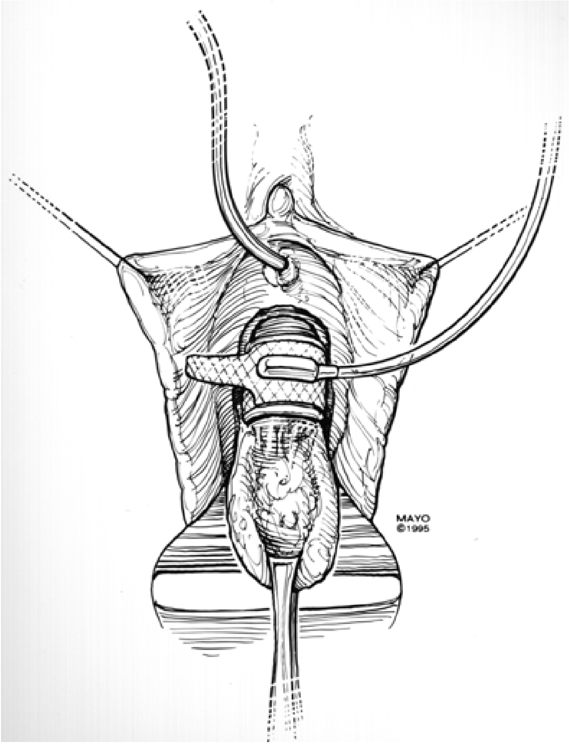

**FIGURE 14.** Completed cuff placement.

### Bladder Neck Cuff Placement in Children

Children who are candidates for AUS implantation are frequently myelodysplastic with urinary incontinence secondary to sphincteric insufficiency and a neurogenic bladder. Many of these children will require intermittent self-catheterization after AUS implantation. They, therefore, should prior to surgery understand the potential outcomes and be able to demonstrate manual dexterity as well as compliance with medical recommendations.

The bladder neck is the only site of cuff implantation in children, both male and female. The preoperative and intraoperative technique follows the same guidelines as for adults with bladder neck cuff placement. The children are positioned supine with knees bent. A lower midline abdominal incision is made and the prevesical space is developed while remaining extraperitoneal. Meticulous dissection must be carried out as the urethral and vaginal tissues are fragile. The plane between the bladder neck and rectum or bladder neck and vagina, respectively, is developed. An anterior midline cystotomy may facilitate dissection. Cuff size in children is usually 6-8cm. A 61-70cm water pressure balloon is used. As with adults, the reservoir is placed in the prevesical space and the pump is placed in either the labia majora or scrotum. To accommodate growth, the pump tubing may need to be lengthened with time. Regular examinations, including voiding cystourethrogram and urodynamic studies, should be completed to avoid upper tract damage.

### AUS Implantation During Bladder Reconstruction

When sphincteric incompetence accompanies detrusor hyperreflexia and low compliance, augmentation cystoplasty combined with AUS implantation can result in continence with a decreased risk of upper tract deterioration. The augmentation cystoplasty is performed first, and then the AUS is implanted (Fig. 15). Cuff placement around the bladder neck follows the same technique as previously described.

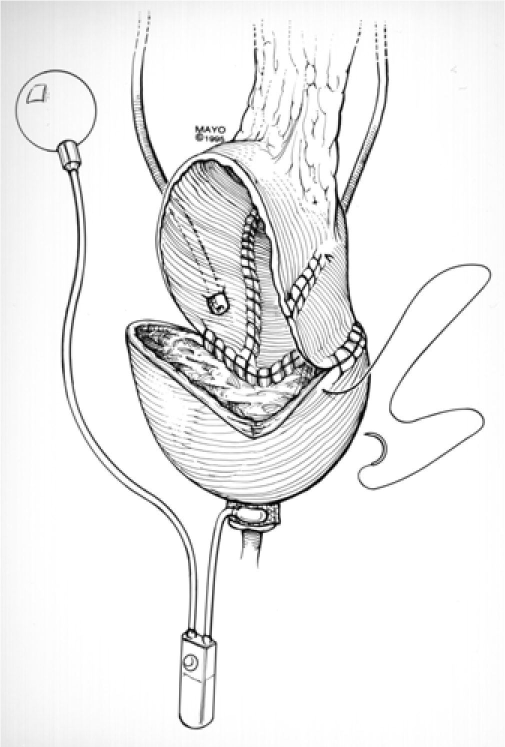

**FIGURE 15.** Augmentation cystoplasty with simultaneous AUS implantation; bladder open to show ureteroneocystotomy.

### POSTOPERATIVE CONSIDERATIONS

Hospital stay after AUS implantation using a bulbous urethral cuff is usually one day with the occasional patient remaining in the hospital for two days. Bladder neck cuff placement requires 4-5 days. Oral pain medication is usually adequate for analgesia. Ice packs applied to the labia or scrotum for 24-48 hours postoperatively reduces swelling around the pump. Broad spectrum intravenous antibiotics (vancomycin and gentamicin) are continued throughout the hospitalization. Oral antibiotics (usually a cephalosporin) are continued for 2 weeks following discharge. Patients may shower daily and take a bath 5 days after surgery.

The Foley catheter is removed the morning after surgery and most patients will be able to urinate. If patients are unable to void, intermittent self-catheterization is begun with a small caliber urethral catheter until spontaneous voiding returns. Prolonged catheterization after AUS implantation should be discouraged due to increased risk of cuff erosion. Some patients will report a transient improvement in urinary control in the immediate postoperative period due to increased resistance from edema near the cuff site. Incontinence is expected during the deactivation period, and, therefore, some form of protection should be worn until sphincter activation.

Patients should be instructed to gently pull down their pump once each day to prevent upward migration during the capsule-forming period. Ambulation is encouraged postoperatively. Patients are cautioned to avoid excessive compression of the perineum and lower abdomen. Heavy lifting and straining is restricted for at least 6 weeks. Patients with bulbourethral cuffs are permanently restricted from riding a bicycle and horseback riding. Sexual activity should be avoided until sphincter activation.

Patients should wear a Medic Alert bracelet to notify medical personnel of the presence of the AUS and the necessity for cuff deactivation prior to Foley catheter placement.

### AUS ACTIVATION

Cuff activation takes place 4-6 weeks following implantation. By this time, scrotal or labial swelling and tenderness has subsided, and incisions are well healed. Successful activation and cuff filling can be confirmed with pre and post-activation radiographs. Patients are given another demonstration of how to cycle the sphincter, and are required to demonstrate, independently, how to compress the pump, open the cuff and void.

Patients who were dry at night prior to sphincter implantation do not require cuff activation for continence while sleeping. These patients can be instructed in cuff deactivation at night. Deactivation has the benefit of reducing the risk of tissue ischemia, urethral atrophy and urethral cuff erosion[5].

If patients are unable to empty their bladder, they are instructed to practice self-intermittent catheterization (SIC) with the cuff deflated during catheterization.

### Immediate Postoperative Complications

Urinary retention may occur in the immediate postoperative period due to urethral edema. The sphincter pump should be checked to confirm that it is in the open position. Most patients will begin voiding after a brief period of intermittent catheterization with a small caliber (10-12F) catheter. If prolonged SIC is required, the cuff should be in the deflated position prior to catheterization. Urinary retention after cuff activation should signal the possibility of stricture disease or bladder neck contracture. Urethral instrumentation should always be performed with the cuff deactivated and with the smallest size cystoscope possible. If prolonged bladder drainage is necessary, a suprapubic tube should be inserted to avoid urethral cuff erosion.

Hematoma is the most common minor complication of AUS implantation. Labial or scrotal hematomas can displace the pump and make external manipulation difficult. Most hematomas will resolve spontaneously. Rarely, a large hematoma will require evacuation.

### Long Term Complications

Persistent or recurrent urinary incontinence is most commonly due to mechanical failure, tissue atrophy or cuff erosion. Inflate-deflate radiographs can evaluate the hydraulic function of the AUS. If the radiopaque fluid is not in the system, then a leak has developed. Surgical exploration and close inspection is required to identify the site of leakage. The most common site of leakage is the lower surface of the cuff.[2] Once the site of leakage has been identified, the individual components can be replaced. If there is fluid present and there is no filling of the cuff during cycling, there is obstruction to flow such as occurs with a tubing kink (a rare occurrence following the availability of kinkless tubing) or foreign body (including air lock) plugging the tubing. Again, surgical exploration is required to identify the location of obstruction. If normal cuff deflation and inflation is present, a urethrogram followed by urethroscopy will exclude cuff erosion. If the onset of the incontinence is insidious, one should suspect cuff-compression tissue atrophy. Urodynamic evaluation with leak point pressure measurement and cystoscopy will reveal poor urethral occlusion when tissue atrophy is present. One of three methods are theoretically available to treat recurrent incontinence due to tissue atrophy: (1) increase the pressure of the balloon reservoir, (2) reduce cuff size by 0.5cm increments, and (3) place a tandem cuff distal to the original cuff in patients with a bulbourethral cuff. In patients with a bladder neck cuff, the reservoir is easier to access than the cuff and therefore the balloon reservoir pressure can be increased. However, in patients with bulbar urethral cuffs, it is never our practice to either increase balloon pressure reservoir or decrease cuff size. It is our feeling that these two procedures will only lead to further worsening of the urethral atrophy and resultant incontinence and possible erosion. Therefore, we managed recurrent incontinence secondary to urethral atrophy with the placement of a tandem cuff.[6]

Cuff erosion is a serious complication requiring cuff removal. Cuff erosion can present with pain, swelling, recurrent incontinence, infection, retention, and bloody urethral discharge. Erosion that presents in the immediate postoperative period is due to an unrecognized iatrogenic urethral injury at the time of surgery. Cystourethroscopy confirms the clinical suspicion. In the absence of infection, the cuff is removed and a stainless steel plug is placed in the cuff tubing. After adequate healing, a new cuff can be inserted. If infection is present, the entire device must be removed. After erosion is identified and the cuff has been removed, a silicone catheter should be left in place for 3-6 weeks to allow for urethral healing. Prosthetic infections can present at anytime but usually will occur within the first 1-2 postoperative months.[2] Following a sufficient recovery time, reoperation and re-implantion of the AUS is encouraged. The surgeon should be aware that this may be a difficult procedure given the degree of reactionary tissue surrounding the bulbar urethra. Currently, we are frequently resorting to transcorporal placement of the cuff to minimize the risk of urethral injury and subsequent erosion[.7] At this point in time, we are very encourage with this approach.

### CONCLUSION

The AUS remains the gold standard for the treatment of stress urinary incontinence in men. Its indications and use in children and woman has decreased over the years secondary to the introduction of better alternatives in these patient populations. It is critical for the urologist implanting the AUS to have a very good working knowledge of the AUS to prevent needless complications and to insure the highest degree of success possible.

### REFERENCES

1. Light, J.K., Implantation of the AS 800 Artificial Urinary Sphincter, Problems in Urology, Vol. 7, Number 3, p402-412, 1993.
2. Elliott, D.S., Barrett, D.M., The Mayo Clinic Long Term Analysis of the Functional Durability of the AMS 800 Artificial Urinary Sphincter: A Review of 323 Cases. Accepted for publication August 1997, J. of Urology.
3. Blum, M.D. Infections of genitourinary prostheses. Infect Dis Clin North Am 1989; 3:259-274.
4. Carson, C.C. III. Infections in genitourinary prostheses. Urol Clin North Am 1989; 16:139-147.
5. Elliott DS, Barrett DM, Gohma M, Boone TB: "Does Nocturnal Deactivation of the Artificial Urinary Sphincter Lessen the Risk for Urethral Atrophy?" Urology, 57 (6) 1051-1054, June 2001.
6. DiMarco DS, Elliott DS: Tandem cuff artificial urinary sphincter as a salvage procedure following failed primary sphincter placement for the treatment of post-prostatectomy incontinence. J. Urol, 170 1252-1254, September 2003.
7. Guralnick ML, Miller E, Toh KL, Webster GD: Transcorporal artificial urinary sphincter cuff placement in cases requiring revision for erosion and urethral atrophy. : J Urol. 2002 May;167(5):2075-8

**This article should be referenced as follows:**

Elliott, D.S. and Barrett, D.M. (2004) The artificial genitourinary sphincter. *TheScientificWorldJOURNAL* **4 (S1)**, 114–127.

**Handling Editor:**

Anthony Atala, Principle Editor for *Urology* — a domain of *TheScientificWorldJOURNAL.*
